# Antibiotic Stewardship in Hospital-Acquired Pneumonia

**DOI:** 10.7759/cureus.73623

**Published:** 2024-11-13

**Authors:** Luqman Khan, Falak Naz, Maria Zeb, Said U Rehman

**Affiliations:** 1 Internal Medicine, Hayatabad Medical Complex, Peshawar, PAK; 2 Gynecology and Obstetrics, Lady Reading Hospital, Peshawar, PAK; 3 Internal Medicine, Medical Teaching Institution-Hayatabad Medical Complex (MTI-HMC), Peshawar, PAK

**Keywords:** antibiotic stewardship, antimicrobial resistance, clinical outcomes, hospital-acquired pneumonia, prospective cohort study

## Abstract

Background

Antimicrobial resistance and incorrect use of antibiotics may worsen hospital-acquired pneumonia (HAP), which is a serious illness associated with healthcare and is related to higher rates of morbidity and death.

Objective

This study aimed to evaluate the effectiveness of antibiotic stewardship programs (ASPs) in optimizing the treatment of HAP, focusing on improving patient outcomes and reducing resistance.

Methodology

A prospective cohort study was conducted from August 2022 to July 2023. Data were gathered on the demographics, comorbidities, antibiotic treatment plans, and clinical outcomes of adult patients with HAP diagnoses. The efficacy of ASPs was evaluated by statistical studies, which included logistic regression.

Results

A total of 428 participants were assessed, with notable differences between the no stewardship group (n = 220, 51.40%) and the stewardship group (n = 208, 48.60%). The stewardship group demonstrated a higher treatment success rate (n = 182, 87.5%) compared to the no stewardship group (n = 126, 57.3%). The 30-day readmission rate was lower in the stewardship group (n = 32, 15.4%) versus the no stewardship group (n = 58, 26.4%), and adverse drug reactions were reduced in the stewardship group (n = 15, 7.2%) compared to the no stewardship group (n = 45, 20.5%). In-hospital mortality was significantly lower in the stewardship group (n = 16, 7.7%) than in the no stewardship group (n = 40, 18.2%).

Conclusion

The results show that ASP implementation greatly improves clinical outcomes for HAP patients, highlighting the need for ongoing funding in ASPs to address antibiotic resistance.

## Introduction

Hospital-acquired pneumonia (HAP) is a serious illness linked to healthcare and often results in longer hospital stays, higher medical expenses, and higher rates of morbidity and death in patients [1,2]. The disorder, which appears at least 48 hours after a patient is admitted to the hospital, is most commonly brought on by organisms that are resistant to multiple drugs as a result of invasive procedures, repeated exposure to the healthcare environment, and the widespread use of broad-spectrum antibiotics [3]. The growing issue of antibiotic resistance makes it more difficult to treat HAP, as it complicates the selection of effective antibiotics. This, in turn, necessitates careful management of antibiotic use to ensure that available resources are used appropriately and to prevent further resistance development [4].

To successfully treat infections while reducing the development of antibiotic resistance, antibiotic stewardship has become apparent as a critical strategy to address these issues [5]. Hospital antibiotic stewardship programs (ASPs) are intended to encourage the proper use of antibiotics by placing a focus on appropriate selection, prompt introduction, and proper dose and duration [6]. The major objective of stewardship is to guarantee that antibiotics continue to be effective against infections over an extended period of time, protecting patient health in the face of developing resistance [7]. Research indicates that by adjusting medication to the narrowest effective range, good antibiotic stewardship may decrease antibiotic use, minimize treatment costs, and enhance patient outcomes [8].

Stewardship is essential in HAP patients because improper or excessive use of antibiotics may develop to resistance and problems that complicate therapy and lengthen hospital stays [9]. According to an increasing amount of evidence, customized stewardship interventions may help HAP patients get better care by educating physicians about best practices, increasing diagnostic precision, and minimizing the needless use of broad-spectrum antibiotics [10,11]. But obstacles such as few resources, inconsistent physician adherence, and difficult diagnostic situations still exist, highlighting the need for ongoing study and stewardship practice optimization [12].

Future policies and actions must be guided by an understanding of how stewardship initiatives may best address issues unique to HAPs. In order to manage HAP, the current research looked at antibiotic stewardship procedures. It assessed their efficacy and identified areas for improvement in hospital settings.

Research objective

This study evaluated the effectiveness of ASPs in treating HAP, identifying specific practices that contributed to optimized patient outcomes and reduced antimicrobial resistance.

## Materials and methods

Study design and setting

This prospective cohort study was conducted at the Hayatabad Medical Complex (HMC), Peshawar, a major tertiary care hospital in Pakistan. The study spanned one year, from August 2022 to July 2023.

Inclusion and exclusion criteria

Adult patients (≥18 years) who were diagnosed with HAP at least 48 hours after admission were included in the research. Individuals who were moved from another medical institution or who had received antibiotic therapy within 48 hours of arrival were not included in the study. To preserve data consistency and reliability, those with incomplete medical records or those diagnosed with non-HAP respiratory illnesses were also eliminated.

Sample size

The sample size for this study was initially calculated using the WHO formula for proportions, yielding a required sample size of 384 participants, based on a 95% confidence level and a 5% margin of error. To account for an anticipated dropout rate of 10%, the adjusted sample size was determined using the formula n (adjusted) = (n/1 - d), resulting in a final target sample size of approximately 428 participants. This adjustment ensures that sufficient data are collected to maintain the statistical power of the study in evaluating the effectiveness of ASPs in treating HAP.

Data collection

Information on the patients' demographics, underlying comorbidities, antibiotic treatment plans, length of antibiotic use, and clinical outcomes (such as length of stay or death) was taken from their medical records. Additionally, information was gathered on antibiotic stewardship initiatives such as duration monitoring, dose modifications, and antibiotic de-escalation. To guarantee consistency, two separate reviewers confirmed the correctness of the data.

Statistical analysis

Continuous data were summarized using frequencies and percentages, whereas categorical variables were given descriptive statistics such as mean and standard deviation. Logistic regression analysis was used to evaluate the impact of ASP therapies on clinical outcomes while controlling for confounding factors such age, comorbidities, and baseline disease severity. P-values less than 0.05 were regarded as statistically significant. Analysis of the data was conducted using SPSS Version 26.0 (IBM Corp., Armonk, NY).

Ethical approval

The Institutional Review Board provided ethical clearance for the research. To maintain anonymity, all patient data were anonymized, and only de-identified data were used for analysis in accordance with institutional and ethical standards.

## Results

The research participants' clinical and demographic details are included in Table 1 for the two groups: the stewardship group (n = 208) and the no stewardship group (n = 220). In the no stewardship group, the mean age was 65.17 ± 11.92 years, whereas in the stewardship group, it was 63.21 ± 12.89 years. Gender breakdown showed that 110 (52.88%) of the stewardship group and 130 (59.09%) of the no stewardship group were men. The frequencies of comorbidities, such as diabetes mellitus (47 [22.59%] vs. 51 [23.18%]) and hypertension (76 [36.54%] vs. 80 [36.36%]), were comparable in both groups. Patients in the no stewardship group were prescribed broad-spectrum antibiotics at a greater rate (150 [68.18%] vs. 118 [56.73%]), and their mean antibiotic use duration was longer (10.48 ± 3.76 days vs. 9.01 ± 3.52 days). The no stewardship group had a substantially higher mortality rate (40 [18.18%] vs. 16 [7.69%]) and a longer mean duration of stay (14.53 ± 5.29 days vs. 10.21 ± 3.82 days). Additionally, the no stewardship group had higher scores for clinical severity indices, such as the Pneumonia Severity Index score (100.21 ± 25.53 vs. 88.42 ± 22.09), SOFA (Sequential Organ Failure Assessment) score (6.84 ± 2.21 vs. 5.23 ± 1.67), and APACHE II (Acute Physiology, Age and Chronic Health Evaluation II) score (20.53 ± 4.52 vs. 17.21 ± 3.82).

**Table 1:** Demographic information and clinical characteristics of study participants APACHE, Acute Physiology, Age and Chronic Health Evaluation; COPD, chronic obstructive pulmonary disease; SOFA, Sequential Organ Failure Assessment

Characteristic		No stewardship group (n = 220)	Stewardship group (n = 208)
Age (years)	Mean ± SD	65.17 ± 11.92	63.21 ± 12.89
Gender, n (%)	Male	130 (59.09)	110 (52.88)
	Female	90 (40.91)	98 (47.12)
Comorbidities, n (%)	Hypertension	80 (36.36)	76 (36.54)
	Diabetes mellitus	51 (23.18)	47 (22.59)
	COPD	41 (18.63)	44 (21.15)
	Heart failure	35 (15.91)	37 (17.79)
	Renal failure	19 (8.63)	15 (7.21)
	Other	10 (4.55)	8 (3.85)
Antibiotic treatment regimens, n (%)	Broad-spectrum antibiotics	150 (68.18)	118 (56.73)
	Combination therapy	70 (31.82)	90 (43.27)
Duration of antibiotic use (days)	Mean ± SD	10.48 ± 3.76	9.01 ± 3.52
Mortality (hospital)	n (%)	40 (18.18)	16 (7.69)
Length of stay (days)	Mean ± SD	14.53 ± 5.29	10.21 ± 3.82
Clinical severity indicators	APACHE II score (mean ± SD)	20.53 ± 4.52	17.21 ± 3.82
	SOFA score (mean ± SD)	6.84 ± 2.21	5.23 ± 1.67
	Pneumonia Severity Index score (mean ± SD)	100.21 ± 25.53	88.42 ± 22.09

Table 2 shows a comparison of the no stewardship group (n = 220) with the stewardship group (n = 208) in terms of antibiotic stewardship strategies and results in controlling HAP. Regarding antibiotic de-escalation, 15 patients (6.82%) in the no stewardship group underwent de-escalation compared to 102 patients (49.04%) in the stewardship group, with a mean time to de-escalation of 6.59 ± 1.87 days and 3.21 ± 1.25 days, respectively. Renal function-based dosage changes were made for 12 patients (5.45%) in the no stewardship group versus 89 patients (42.79%) in the stewardship group; body weight-based adjustments were made for eight (3.64%) patients in the no stewardship group compared to 66 patients (31.73%) in the stewardship group. The stewardship group exhibited a significantly higher adherence rate to recommended duration guidelines, with 50 (22.73%) patients in the no stewardship group and 172 (82.69%) patients in the stewardship group. Additionally, the average duration of antibiotic therapy was lower in the stewardship group (9.01 ± 3.52 days) than in the no stewardship group (10.48 ± 3.76 days). Clinical results demonstrated a significant difference, with treatment success (resolution of infection) in the no stewardship group in 126 (57.27%) patients and in the stewardship group in 182 (87.50%) patients. The incidence of adverse medication responses decreased dramatically from 45 (20.45%) patients in the no stewardship group to 15 (7.21%) patients in the stewardship group, and the 30-day readmission rate was lower in the stewardship group, with 32 (15.38%) patients compared to 58 (26.36%) patients in the no stewardship group.

**Table 2:** Antibiotic stewardship interventions and outcomes in the management of hospital-acquired pneumonia

Intervention category		No stewardship (n = 220)	Stewardship (n = 208)
Antibiotic de-escalation	De-escalation performed	15 (6.82%)	102 (49.04%)
	Time to de-escalation (days, mean ± SD)	6.59 ± 1.87	3.21 ± 1.25
Dosage adjustments	Adjusted dosage based on renal function	12 (5.45%)	89 (42.79%)
	Adjusted dosage based on body weight	8 (3.64%)	66 (31.73%)
Duration monitoring	Adherence to recommended duration guidelines, n (%)	50 (22.73%)	172 (82.69%)
	Average duration of antibiotic therapy in days (mean ± SD)	10.48 ± 3.76	9.01 ± 3.52
Clinical outcomes	Treatment success (resolution of infection)	126 (57.27%)	182 (87.50%)
	30-day readmission rate	58 (26.36%)	32 (15.38%)
	Incidence of adverse drug reactions	45 (20.45%)	15 (7.21%)

Table 3 shows a comparison of the no stewardship group (n = 220) with the stewardship group (n = 208), illustrating the impact of antibiotic stewardship treatments on clinical outcomes. The stewardship group had a considerably better treatment success rate, with 182 (87.50%) patients achieving resolution of infection compared to 126 (57.27%) patients in the no stewardship group, resulting in an odds ratio of 4.10 (95% CI: 2.56-6.57) and a p-value of less than 0.001. Additionally, the 30-day readmission rate was lower in the stewardship group, with 32 (15.38%) patients compared to 58 (26.36%) patients in the no stewardship group, yielding an odds ratio of 0.50 (95% CI: 0.31-0.81) and a p-value of 0.005. Furthermore, the incidence of adverse medication responses was significantly lower in the stewardship group, with 15 (7.21%) patients versus 45 (20.45%) patients in the no stewardship group, as indicated by an odds ratio of 0.29 (95% CI: 0.16-0.53) and a p-value of <0.001. The in-hospital death rate was also lower in the stewardship group, with 16 (7.7%) patients compared to 40 (18.2%) patients in the no stewardship group, leading to an odds ratio of 0.35 (95% CI: 0.18-0.68) and a p-value of 0.003, indicating a noteworthy decrease in mortality linked to antimicrobial stewardship initiatives.

**Table 3:** Effectiveness of antibiotic stewardship interventions on clinical outcomes

Clinical outcome	No stewardship (n = 220)	Stewardship (n = 208)	Odds ratio (95% CI)	p-Value
Treatment success (resolution of infection)	126 (57.27%)	182 (87.50%)	4.10 (2.56-6.57)	<0.001
30-day readmission rate	58 (26.36%)	32 (15.38%)	0.50 (0.31-0.81)	0.005
Incidence of adverse drug reactions	45 (20.45%)	15 (7.21%)	0.29 (0.16-0.53)	<0.001
Mortality (in-hospital)	40 (18.2%)	16 (7.7%)	0.35 (0.18-0.68)	0.003

## Discussion

The study's findings show that ASPs have a big influence on how HAP is managed. According to our research, the treatment success rate was 87.50% in the stewardship group whereas it was just 57.27% in the no stewardship group (p < 0.001). This is consistent with other studies that have shown that the use of ASPs may result in better clinical results, such as increased treatment success rates for patients with HAP [13,14]. Our study's enhanced infection resolution highlights the significance of customized antibiotic treatments, especially in light of the rising incidence of multidrug-resistant bacteria in hospital environments.

Additionally, the research demonstrated that the stewardship group's 30-day readmission rate (15.38%) was much lower than that of the no stewardship group (26.36%) (p = 0.005). This result is consistent with other research, which found that good stewardship initiatives improve overall patient management in addition to lowering readmission rates [15]. Better adherence to advised treatment recommendations and the proactive management of antibiotic therapies - a key component of successful ASPs - can be credited with the decrease in readmissions.

Furthermore, our data show a significant drop in adverse medication responses, with a rate of just 7.21% in the stewardship group and 20.45% in the no stewardship group (p < 0.001). This notable distinction is consistent with earlier research, which highlighted how ASPs, by selecting the right antibiotics and adjusting dosages, might reduce the likelihood of adverse medication events [16]. Our findings imply that a methodical approach to the prescription of antibiotics optimizes patient safety by lowering the frequency of side effects related to antibiotic treatments, in addition to improving therapeutic results.

Another important finding of our research is the decrease in in-hospital mortality rates, with 7.7% mortality in the stewardship group and 18.2% in the no stewardship group (p = 0.003). These findings are in line with earlier research, which showed that HAP patients who get effective ASPs had reduced death rates [17]. Empirical data indicate that the adoption of stewardship measures might result in a noteworthy reduction in mortality by means of enhanced antibiotic use and administration, hence improving patient outcomes.

Strength and limitations

Significant aspects of this research include a well-defined patient group, thorough data collection techniques, and the use of strong statistical analysis to assess how successfully ASPs cure HAP. The obvious differences between the stewardship and non-stewardship groups demonstrate the measurable advantages of ASPs, including higher rates of treatment success and lower rates of adverse responses. Nevertheless, there are several drawbacks, such as the single-center approach, which might restrict how broadly the results can be applied, and possible biases connected to physicians' compliance with stewardship guidelines. Furthermore, the study's observational design makes it more difficult to conclusively prove causality. These elements highlight the need for more multi-center research to confirm the findings and improve knowledge of the efficacy of ASP in various healthcare environments.

## Conclusions

This research emphasizes how crucial ASPs are to efficiently control HAP. The stewardship group's notable advancements in treatment success rates, 30-day readmission rates, adverse drug reactions, and in-hospital mortality show that customized interventions can significantly improve patient outcomes while lowering the risks related to antibiotic misuse. These results validate the use of strong ASPs in hospital environments to address the problems caused by multidrug-resistant pathogens and to maximize antibiotic use. In the end, our findings highlight the need for continued funding and dedication to antibiotic stewardship, which is critical for protecting patient safety and guaranteeing the effectiveness of antibiotics for future generations.
